# The Impact of COVID-19 on the Obstetrics and Gynecology Clinical Clerkship Experience

**DOI:** 10.7759/cureus.78572

**Published:** 2025-02-05

**Authors:** Brianne Yarranton, Hava Starkman, Anton Svendrovski, Thirushi Siriwardena, Dini Hui, Carmen McCaffrey

**Affiliations:** 1 Obstetrics and Gynecology, Temerty Faculty of Medicine, University of Toronto, Toronto, CAN; 2 Statistics, UZIK Consulting Inc, Toronto, CAN

**Keywords:** clinical clerkship, covid-19, obstetrics and gynecology (ob/gyn), undergraduate and graduate medical education, undergraduate medical student

## Abstract

Background

The COVID-19 pandemic disrupted many aspects of medical education; however, there is currently no published literature describing the pandemic’s impact on the obstetrics and gynecology (OBGYN) clerkship experience. The purpose of this study was to survey medical students at the University of Toronto who completed their OBGYN rotation during the pandemic to gain perspective on how it impacted their clerkship experience.

Methods

An anonymous, voluntary survey regarding the OBGYN clerkship experience was circulated to all University of Toronto medical students who completed their clerkships between September 2020 and August 2021. Data was collected between December 3, 2021 and January 3, 2022.

Results

Of the 255 students surveyed, 95 (36.4%) responded. Among them, 57 (64%) reported increased stress during their clerkship, while 40 (44.9%) found their OBGYN rotation to be more stressful than other rotations. Additionally, 30 (33.7%) indicated that the pandemic led them to question their choice of medicine as a career. Regarding the quality of their OBGYN rotation, 21 (24.4%) noticed a significant change, with 29 (33.7%) attributing this to a lack of patients and 35 (40.7%) expressing concerns about acquiring hands-on skills. A thematic analysis of student responses identified four key themes: Virtual Learning, Clinical Workload and Volume, Surgical Cancellations, and Caring for Patients During COVID.

Conclusions

The pandemic has had a significant impact on the OBGYN clerkship experience and student well-being. This study highlights several quality concerns, including reduced clinical volumes and limited hands-on learning opportunities, particularly in gynecology.

## Introduction

The COVID-19 pandemic has had an unprecedented impact on many aspects of the Canadian medical system, including medical education across the country and around the world. Several studies have been conducted to investigate the impact on medical trainees, including medical clerks and residents. To date, a number of studies have documented the mental health impacts experienced by medical clerks during the pandemic, including increased anxiety and depression [1,2]. As it relates to medical training, studies demonstrate that medical clerks report increased stress levels related to decreased opportunities to match to residency, concerns about preparation for national licensing exams, and the effect of the resulting disruption to the academic calendar [3-5]. One survey of medical students demonstrated that many felt concerned about their risk of contracting COVID-19 during clerkship; however, most students reported overall positive experiences [6]. Studies investigating the pandemic’s effect on resident training and well-being also demonstrated similarly elevated anxiety levels regarding issues such as the availability of personal protective equipment and staffing issues in clinics [7,8].

Within the Canadian medical school system, the obstetrics and gynecology (OBGYN) clerkship rotation normally includes exposure to labor and delivery, gynecologic surgery, and general OBGYN clinics. This rotation places emphasis on exposure to common clinical encounters in OBGYN, including abnormal uterine bleeding, medical care during pregnancy, contraception counseling, and pelvic pain. Students also receive exposure to hands-on procedures such as speculum insertion, prenatal exams, and spontaneous vaginal deliveries. The goal of this rotation is to ensure access to opportunities to learn skills and knowledge that any physician should have, regardless of the specialty they enter. Given the hands-on nature of the specialty, it can be especially challenging to teach these skills virtually. While educators have endeavored to maintain the same quality of teaching and clinical exposure during the pandemic, it is unclear what impact pandemic-related changes have had on the experience of undergraduate medical students completing this clerkship rotation.

Unfortunately, there is currently no research examining the impact of the COVID-19 pandemic on the clinical learning experience for medical students during their OBGYN clerkship rotation. The goal of this study was to survey undergraduate medical students at the University of Toronto who completed their OBGYN rotation during the COVID-19 pandemic to gain student perspectives on how their education was impacted. This study had three primary research questions: (1) How has the COVID-19 pandemic impacted the clinical learning experience of undergraduate medical students undertaking their OBGYN clerkship rotation at the University of Toronto? (2) What are the various stressors identified by undergraduate medical students during their OBGYN rotation throughout the COVID-19 pandemic? (3) How are undergraduate medical students utilizing university support systems and other resources as solutions to these pandemic stressors during their OBGYN rotation? 

This article was previously presented as an abstract at the 2022 AMEE (Association for Medical Education in Europe - International Association for Health Professions Education) Hybrid Conference on August 27, 2022.

## Materials and methods

Study design

This study was approved by the University of Toronto Research Ethics Board (protocol #29245). Informed consent was obtained from each survey participant. All methods were performed in accordance with the relevant guidelines and regulations. This is a cross-sectional study in which an electronic survey was distributed to medical students (n= 255) who had completed their OBGYN clerkship rotation during the COVID-19 pandemic via a commercially available survey platform (Survey Monkey). Responses were monitored to ensure each response was generated from a unique student and that multiple responses were not generated from a single participant.

Inclusion criteria included fourth-year medical students from the University of Toronto's undergraduate medical program who completed their OBGYN core clerkship rotation between September 2020 and August 2021. Exclusion criteria encompassed students who did not complete their OBYGYN core clerkship during this period or did not complete their rotation at the University of Toronto.

Data collection

The survey included 28 questions in English on the topic of the OBGYN clerkship experience. The survey was developed based on previously published surveys from similar studies [4,6,7] as well as the WHO Well-Being Index [9]. The survey included both open- and closed-ended questions and was divided into five separate sections, including demographic information, stress, quality of rotation, well-being, and stress management and resources. Two questions had a five-point Likert format. A copy of the survey can be found in Appendix A. Participation was voluntary and anonymous, and a $5 gift card was provided to every student who completed the survey. The survey was distributed via email and a private Facebook group to all eligible participants between December 3, 2021 and January 3, 2022. Two reminders were sent to students during the study enrollment period.

Statistical analysis

Statistical analysis was conducted using IBM SPSS Statistics for Windows, Version 28.0 (Released 2021; IBM Corp., Armonk, NY, USA). Quantitative survey responses were summarized using descriptive statistics (frequency/percentages for categorical data and mean/SD for numerical variables). The association between numerical variables was assessed using Pearson correlation analysis. Comparisons of responses between three or more categories for numerical variables were compared using one-way ANOVA and for two groups using independent-sample t-tests. The Welch version of the t-test was used in situations with unequal variances. Inferential statistical analysis was based on a level of significance of 0.05, with p-values <0.05 reported as statistically significant.

The internal reliability for Likert-scale questions was measured using Cronbach’s alpha, with a value of >0.8 suggesting an appropriately high level of reliability. For questions with high internal reliability, a composite score was calculated using the mean of the category responses.

Qualitative analysis

Content analysis of the qualitative survey responses was completed using NVivo software. Responses were coded and categorized into themes independently by two authors (BY and HS), with participant quotations chosen to illustrate identified themes. After the completion of independent analysis, themes were compared and verified between the two investigators to reach a consensus on the interpretation of the data [10].

## Results

The survey was completed at least partially by 93 participants out of the 255 students (36.4%) to whom the survey was distributed.

Demographics

Table 1 summarizes the demographic information collected from participants. A total of 58 (62.4%) respondents identified as female, with 32 (34.4%) identifying as male, and one (1.1%) and two (2.2%) identifying as another gender identity or preferred not to disclose. Moreover, 16 (17.2%) students were considering a career in OBGYN prior to their OBGYN clerkship rotation.

**Table 1:** Demographic and professional characteristics (n = 93) OBGYN, obstetrics and gynecology

Characteristics	Frequency (%) or mean ± SD {range}
Age (years)	26.09 ± 4.01 {23-56}
Gender	
Female	58 (62.4%)
Male	32 (34.4%)
Other	1 (1.1%)
Prefer not to say	2 (2.1%)
Considered a career in OBGYN prior to rotation	16 (17.2%)

Stress and pandemic impact 

Table 2 summarizes responses to questions regarding their stress level during their OBGYN clerkship rotation. Of these responses, 64% (57/93) of students reported increased stress levels during clerkship, while 44.9% (40/93) reported increased stress levels during their OBGYN rotation and 47.2% (42/93) reported stress levels were similar to those experienced in other rotations. Only three (3.4%) students experienced an increased interest in pursuing OBGYN as a specialty. Furthermore, 33.7% (30/93) of students questioned their choice of medicine as a career because of the COVID-19 pandemic.

**Table 2:** Stress and pandemic impact (n = 89) OBGYN, obstetrics and gynecology

Stress and pandemic impact categories	Frequency (%), n = 89
Stress level during clerkship within the context of the COVID-19 pandemic	
Higher than usual	57 (64.0%)
Similar to usual	29 (32.6%)
Lower than usual	3 (3.4%)
Stress level during OBGYN clerkship rotation within the context of the COVID-19 pandemic	
Higher than other rotations	40 (44.9%)
Similar to other rotations	42 (47.2%)
Lower than other rotations	7 (7.9%)
Reconsidered the choice of the residency program as a result of the current pandemic	11 (12.4%)
Have a greater interest in OBGYN	3 (3.4%)
Have a lesser interest in OBGYN	8 (9.0%)
Question choice of medicine as a career as a result of the current pandemic	30 (33.7%)

Table 3 summarizes responses to questions regarding the various stressors specific to the OBGYN clerkship rotation. “Fear of contracting COVID-19 or of contaminating someone else” was the most stressful factor (mean Likert score = 2.74 ± 1.19), and “Suspension of OBGYN clerkship rotations” was the least stressful factor (mean Likert score = 1.66 ± 1.13). Table 4 summarizes the correlation of stress scores for each of the factors that participants were surveyed about. A composite stress score was calculated using the mean of nine-factor scores, and ranged from 0 to 4.22, with a mean value of 2.28 and an SD of 1.01.

**Table 3:** Factors impacting stress during the OBGYN rotation within the context of the COVID-19 pandemic (n = 89) ^1^ Scores range from 0 to 5, with 0 being not stressful and 5 being an extreme level of stress CaRMS, Canadian Resident Matching Service; OBGYN, obstetrics and gynecology

Factors	Mean ± SD score^1^
Fear of contracting COVID-19 or of contaminating someone else	2.74 ± 1.19
Suspension of OBGYN clerkship rotations	1.66 ± 1.13
Modalities of rotations (e.g., lack of supervision and lack of learning opportunities)	2.12 ± 0.99
CaRMS applications	2.45 ± 1.54
Feeling the need to use this time to be productive in view of CaRMS	2.69 ± 1.61
Not having enough letters of recommendation or having letters of poorer quality	2.16 ± 1.40
CaRMS application deadline	2.07 ± 1.40
Cancellation of electives or not being able to book preferred electives	2.33 ± 1.34
Not being able to complete electives outside of home university	2.30 ± 1.46

**Table 4:** Correlations of stress scores between factors impacting OBGYN rotation (n = 89) Significant correlations at * p < 0.05, ** p < 0.01, and *** p < 0.001, CaRMS, Canadian Resident Matching Service; OBGYN, obstetrics and gynecology

Factors	-1	-2	-3	-4	-5	-6	-7	-8
(1) Fear of contracting COVID-19 or of contaminating someone else								
(2) Suspension of OBGYN clerkship rotations	0.29**							
(3) Modalities of rotations (e.g., lack of supervision and lack of learning opportunities)	0.44***	0.45***						
(4) CaRMS applications	0.37***	0.46***	0.42***					
(5) Feeling the need to use this time to be productive in view of CaRMS	0.47***	0.34***	0.33**	0.78***				
(6) Not having enough letters of recommendation or having letters of poorer quality	0.32**	0.49***	0.41***	0.73***	0.74***			
(7) CaRMS application deadline	0.35***	0.49***	0.36***	0.79***	0.75***	0.75***		
(8) Cancellation of electives or not being able to book preferred electives	0.2	0.45***	0.35***	0.45***	0.41***	0.59***	0.50***	
(9) Not being able to complete electives outside of home university	0.26*	0.48***	0.31**	0.63***	0.55***	0.64***	0.71***	0.64***

Quality of rotation

Table 5 summarizes responses regarding the impact of COVID-19 on the quality of the educational experience during their rotation. A total of 21 (24.4%) participants felt that the pandemic had caused a significant impact on the quality of their rotation.

**Table 5:** Impact of COVID-19 pandemic on OBGYN rotation (n = 86) OBGYN, obstetrics and gynecology; PPE, personal protective equipment

Impact categories	Frequency (%)
Felt a noticeable change in the quality of OBGYN rotation due to the COVID-19 pandemic	21 (24.4%)
Experienced a lack of patients in the clinic due to the COVID-19 pandemic	29 (33.7%)
Felt experience was impacted by providing virtual care vs. in-person care	27 (31.4%)
Felt limited in ability to learn hands-on skills (cervical exams, assisting in vaginal deliveries, etc.) due to the COVID-19 pandemic	35 (40.7%)
Experienced reduced opportunities to participate in surgical procedures due to the COVID-19 pandemic	26 (30.2%)
Experienced a lack of PPE at any point during the rotation	1 (1.2%)
Felt learning experience was affected by the provision of OBGYN teaching seminars in a virtual fashion rather than in-person	15 (17.4%)

Well-being

Table 6 summarizes responses to the WHO Well-Being Index. The overall mean WHO Well-Being Index score for participants who responded was 38.51 ± 22.11. Table 7 summarizes the correlations between the well-being categories during the OBGYN rotation. A composite well-being score was calculated using the mean of five category scores, and ranged from 0 to 5, with the mean value 1.93 and SD 1.10. We found no statistically significant correlation between composite stress and composite well-being scores, r(86) = -0.08, p = 0.44.

**Table 6:** Overall well-being during OBGYN rotation (WHO Well-being Index, n = 86) OBGYN, obstetrics and gynecology

Well-being category (feeling a particular emotion)	Numeric score (0-5), mean ± SD	Frequency of feeling					
		At no time (0)	Some of the time (1)	Less than half of the time (2)	More than half of the time (3)	Most of the time (4)	All the time (5)
Cheerful and in good spirits	2.28 ± 1.28	8 (9.3%)	19 (22.1%)	17 (19.8%)	26 (30.2%)	15 (17.4%)	1 (1.2%)
Calm and relaxed	1.93 ± 1.30	13 (15.3%)	23 (27.1%)	17 (20.0%)	22 (25.9%)	9 (10.6%)	1 (1.2%)
Active and vigorous	2.04 ± 1.30	13 (15.3%)	18 (21.2%)	19 (22.4%)	24 (28.2%)	10 (11.8%)	1 (1.2%)
Woke up fresh and rested	1.42 ± 1.21	22 (25.6%)	29 (33.7%)	18 (20.9%)	12 (14.0%)	4 (4.7%)	1 (1.2%)
Daily life was filled with interesting things	2.01 ± 1.21	7 (8.1%)	29 (33.7%)	17 (19.8%)	23 (26.7%)	9 (10.5%)	1 (1.2%)

**Table 7:** Correlations of well-being categories during OBGYN rotation (n = 86) Significant correlations at * p < 0.05, ** p < 0.01, and *** p < 0.001 OBGYN, obstetrics and gynecology

Well-being category	-1	-2	-3	-4
(1) Cheerful and in good spirits				
(2) Calm and relaxed	0.78***			
(3) Active and vigorous	0.78***	0.74***		
(4) Woke up fresh and rested	0.65***	0.71***	0.63***	
(5) Daily life was filled with interesting things	0.79***	0.64***	0.74***	0.57***

Stress management and resources

Table 8 summarizes responses to questions regarding use of university mental health and wellness resources. Sixty-one (72.6%) students did not use university resources and only 14 (16.7%) students used university resources and found them helpful. Twenty-one (24.4%) students indicated that they felt these resources were easily accessible.

**Table 8:** Use of university resources (n = 86)

	Frequency (%)
Use of university resources to overcome difficult moments during the COVID-19 pandemic	
Yes, used university resources and I found them helpful	14 (16.7%)
Yes, used university resources, but I did not find them helpful	9 (10.7%)
No, I did not use any university resources	61 (72.6%)
University resources used (n = 23)	
Student support network	12 (52.2%)
Student association	3 (13.0%)
Members of your faculty	4 (17.4%)
Support line	2 (8.7%)
Mobile applications or other online resources	2 (8.7%)
Find university resources relating to stress and mental health easy to access during the COVID-19 pandemic	
Yes, university resources were easily accessed	21 (24.4%)
No, university resources were difficult to access	12 (14.0%)
Did not use university resources during the COVID-19 pandemic	53 (61.6%)

Interest in OBGYN

Participants were divided into two groups based on whether they endorsed an interest in OBGYN prior to the start of the rotation and the comparison of these groups is summarized in Table 9. Respondents who had considered a career in OBGYN prior to their rotation reported significantly higher stress levels compared to those who did not.

**Table 9:** Comparison between two subgroups regarding career in OBGYN (n = 89) ^1^ Welch t-test was used as the assumption of equal variances violated. ^2^ The composite well-being score is calculated by summing the responses to five questions and multiplying the total by four, resulting in a theoretical range of 0-100. CaRMS, Canadian Resident Matching Service; OBGYN, obstetrics and gynecology

	Considered a career in OBGYN prior to rotation		Comparison test
	Yes (n = 16)	No (n = 73)	
Factors impacting OBGYN rotation			
Fear of contracting COVID-19 or of contaminating someone else	3.06 ± 1.18	2.67 ± 1.19	t(87) = 1.19, p = 0.24
Suspension of OBGYN clerkship rotations	2.31 ± 1.35	1.52 ± 1.03	t(87) = 2.63, p = 0.01
Modalities of rotations (e.g., lack of supervision and lack of learning opportunities)	2.63 ± 0.96	2.01 ± 0.96	t(87) = 2.30, p = 0.02
CaRMS applications	2.88 ± 1.36	2.36 ± 1.57	t(87) = 1.23, p = 0.22
Feeling the need to use this time to be productive in view of CaRMS	2.94 ± 1.34	2.63 ± 1.67	t(26.3) = 0.79^1^, p = 0.44
Not having enough letters of recommendation or having letters of poorer quality	2.75 ± 1.34	2.03 ± 1.38	t(87) = 1.90, p = 0.06
CaRMS application deadline	2.13 ± 1.26	2.05 ± 1.43	t(87) = 0.18, p = 0.86
Cancellation of electives or not being able to book preferred electives	3.06 ± 1.12	2.16 ± 1.33	t(87) = 2.50, p = 0.01
Not being able to complete electives outside of home university	2.81 ± 1.33	2.19 ± 1.47	t(87) = 1.56, p = 0.12
Composite stress score	2.73 ± 0.81	2.18 ± 1.02	t(87) = 2.00, p = 0.048
Well-being category			
Cheerful and in good spirits	2.69 ± 1.08	2.19 ± 1.31	t(84) = 1.42, p = 0.16
Calm and relaxed	1.94 ± 1.00	1.93 ± 1.36	t(83) = 0.03, p = 0.98
Active and vigorous	2.13 ± 1.09	2.01 ± 1.36	t(83) = 0.30, p = 0.76
Woke up fresh and rested	1.25 ± 0.58	1.46 ± 1.32	t(54.9) = -0.97^1^, p = 0.34
Daily life was filled with interesting things	2.19 ± 1.11	1.97 ± 1.24	t(84) = 0.64, p = 0.52
Composite well-being score^2^	40.75 ± 15.54	38.00 ± 23.41	t(84) = 0.45, p = 0.66

Reconsideration of a career in medicine

Participants were divided into two groups based on whether they reported reconsidering their choice of a career in medicine based on the pandemic. The comparison of the two subgroups is summarized in Table 10. There were significantly higher reported stress levels and lower well-being scores among participants who indicated that they had questioned their choice of medicine as a career because of the pandemic (p < 0.05). Those participants who questioned their choice of medicine as a career also had significantly lower WHO Well-Being Index scores.

**Table 10:** Comparison between two subgroups questioning the choice of medicine as a career (n = 89) ^1^ Welch t-test was used as the assumption of equal variances violated. ^2^ The composite well-being score is calculated by summing the responses to five questions and multiplying the total by four, resulting in a theoretical range of 0-100. CaRMS, Canadian Resident Matching Service; OBGYN, obstetrics and gynecology

	Question choice of medicine as a career as a result of the current pandemic		Comparison test
	Yes (n = 30)	No (n = 59)	
Factors impacting OBGYN rotation			
Fear of contracting COVID-19 or of contaminating someone else	3.20 ± 1.13	2.51 ± 1.17	t(87) = 2.68, p = 0.01
Suspension of OBGYN clerkship rotations	1.90 ± 1.12	1.54 ± 1.12	t(87) = 1.42, p = 0.16
Modalities of rotations (e.g., lack of supervision and lack of learning opportunities)	2.43 ± 1.19	1.97 ± 0.83	t(43.7) = 1.92^1^, p = 0.06
CaRMS applications	2.93 ± 1.64	2.20 ± 1.44	t(87) = 2.16, p = 0.03
Feeling the need to use this time to be productive in view of CaRMS	3.20 ± 1.65	2.42 ± 1.54	t(87) = 2.19, p = 0.03
Not having enough letters of recommendation or having letters of poorer quality	2.53 ± 1.50	1.97 ± 1.31	t(87) = 1.83, p = 0.07
CaRMS application deadline	2.47 ± 1.55	1.86 ± 1.28	t(49.7) = 1.84^1^, p = 0.07
Cancellation of electives or not being able to book preferred electives	2.70 ± 1.26	2.14 ± 1.34	t(87) = 1.91, p = 0.06
Not being able to complete electives outside of home university	2.73 ± 1.48	2.08 ± 1.41	t(87) = 2.02, p = 0.046
Composite stress score	2.68 ± 0.95	2.08 ± 0.98	t(87) = 2.75, p = 0.01
Well-being category			
Cheerful and in good spirits	1.79 ± 1.24	2.53 ± 1.24	t(84) = -2.59, p = 0.01
Calm and relaxed	1.43 ± 1.23	2.18 ± 1.27	t(83) = -2.58, p = 0.01
Active and vigorous	1.76 ± 1.43	2.18 ± 1.22	t(83) = -1.42, p = 0.16
Woke up fresh and rested	1.00 ± 1.04	1.63 ± 1.25	t(84) = -2.34, p = 0.02
Daily life was filled with interesting things	1.45 ± 1.06	2.30 ± 1.19	t(84) = -3.24, p < 0.01
Composite well-being score^2^	29.52 ± 21.28	43.09 ± 21.26	t(84) = -2.80, p = 0.01

Qualitative analysis

After completing the thematic analysis for qualitative responses provided by students, it was possible to identify four different theme,: Virtual Learning, Clinical Workload and Volume, Surgical Cancellations, and Caring for Patients During COVID, as summarized in Table 11.

**Table 11:** Identified themes, subcategories, and sample quotes from qualitative analysis of responses CaRMS, Canadian Resident Matching Service; OBGYN, obstetrics and gynecology

Theme:	Subcategory	Sample quote
Virtual Learning	Positive Aspects	“It {virtual learning} was actually helpful and more accessible, e.g., on post-call days.”
		“Online rounds allowed us all take part in the experience despite being at different centers.”
	Negative Aspects	“I always prefer in-person learning - I feel it is a better method of knowledge transmission as is more ‘present,’ attentive, and you can learn from verbal and nonverbal cues from the instructor in addition to presentation aids, etc.”
		“…did not get a chance to ask questions in-person and perhaps lost out on more procedural training.”
		“Since we weren’t there in person for teaching, I felt like there was a great expectation to attend virtual teaching since it is accessible by phone even though I may have been at work. Sometimes I would be in the hospital but not specifically dismissed to attend teaching.”
Clinical Workload and Volume	Decreased Clinical Volumes – Positive Aspects	“Shortened 26-hour call shifts into manageable eight- to 12-hour shifts.”
		“We had pre-call days and post-call days which allowed me to study more and have self-care.”
		“More study time when clinics fell through.”
	Decreased Clinical Volumes – Negative Aspects	“Fewer patients so less clinical learning and reduced opportunity for feedback or for reference letters.”
		“Very little opportunities to do routine prenatal checkups, pelvic/cervical exams, Leopold’s maneuvers, and birth control checkups.”
		“Less opportunity to participate in clinics such as colposcopy. Less opportunity to see patients in person to perform spec (speculum) exams, insert IUDs, EMBx (endometrial biopsy), etc.”
		“Little understanding of gyne concerns. Barely any clinic experience regarding gyne complaints.”
	Virtual Care	“Bread and butter cases which are good learning opportunities for year 3 were mostly virtual; basic Paps, bimanual exams, etc., were hard to come by.”
		“I am interested in specializing in family medicine. I had hoped that my OBGYN rotation would teach me skills necessary to primary care such as conducting prenatal check-ups, providing birth control, doing pelvic exams, and doing pap smears. However, because of the COVID-19 pandemic many of the outpatient OBGYN clinics have transitioned to mostly virtual care and were not taking medical students.”
Surgical Cancellations		“OR volumes were low; I saw only two surgical procedures (besides emergency C-sections) during my rotation.”
		“ORs were often being canceled (often the day before or the week before) because of the second wave of the pandemic.”
Caring for Patients During COVID	Positive Aspects	“We had the opportunity to book pregnant patients for expedited vaccination as it had just become available. Answering their questions and knowing we were doing something to protect them was very meaningful.”
		“It was interesting to see COVID-19 cases in pregnancy and how those were managed, and it was helpful to do counseling around the vaccine in pregnancy.”
		“Less outside people in the labor unit making some interactions easier with patients and their families.”
	Negative Aspects	“The masks of course make communication difficult in terms of picking up nonverbal cues and facial expressions.”
		“Less visitors for patients … made it harder to provide sensitive care.”
		“Removed a lot of the joy from the L&D (labor and delivery) ward with no visitors.”

## Discussion

The COVID-19 pandemic has had a significant impact on the mental health and well-being of medical students completing their OBGYN clerkship rotations. The survey responses outlined that 64% of students experienced increased stress levels during clerkship because of the COVID-19 pandemic, which echoes the finding of other studies that have found increased stress levels in medical students during the pandemic [3-5]. Most students considered the OBGYN rotation to be as stressful or more so than other rotations. This highlights a need for greater support for students during this rotation and should be kept in mind for those teaching medical students during this rotation.

Approximately one-third of students surveyed reported that the pandemic had caused them to reconsider their choice of a career in medicine. This is higher than reported by Abbas et al., in which 19% of students reconsidered their choice of medicine [4], as compared to 33.7% of students in our study. When considering student responses to the WHO Well-Being Index, respondents outlined that despite higher reported stress scores, they had improved overall Well-Being Scores, with a mean of 38.51, compared to that reported by Abbas et al., of 14.8 [4]. This response rate finding, while different from that in the Abbas et al. study, is congruent with studies finding a lack of correlation between stress and well-being scores. The previous finding that students who reported reconsidering a career in medicine had significantly higher stress scores and lower well-being scores [4] was also confirmed in this study.

Students with a prior interest in pursuing a career in OBGYN were also found to have significantly higher stress scores than those who were not. Clerkship rotations present the opportunity to network with clinical supervisors and obtain letters of reference for the upcoming residency application process. It is reasonable to expect that those students interested in a particular specialty might experience higher stress related to their performance and these opportunities for career advancement, especially when exposure to clinical opportunities is more limited.

Of particular concern, this study has reflected other literature suggesting that while the pandemic has had significant impacts on mental health and well-being, the majority of medical students are not making use of university resources. Although the proportion of students in this study who reported using these resources (27.4%) was higher than previously reported (11.7%) [4], nearly a quarter of the students in our study indicated that they did not feel that their university and medical school mental health and wellness resources were accessible. The poor utilization of mental health resources reinforces the need for effective, accessible mental health and wellness resources for medical students and highlights the need to ensure that students are aware of these resources if they do exist.

Another theme identified was reduced clinical volumes. One of the predominant issues that students identified was a challenge in acquiring important hands-on experience in skills such as speculum exams and cervical assessments that are integral to the OBGYN rotation experience. Of the participants, 35 (40.7%) indicated that the pandemic limited their ability to learn these hands-on skills. Many students felt they missed out on valuable gynecology experience by virtue of surgical cancellations and virtual care. The default when there were issues finding learning opportunities for students seemed to be to place students in labor and delivery wards or in obstetrical clinics where they would be more likely to see patients in person. As a result, students felt they did not receive sufficient exposure to gynecology to feel comfortable with the clinical skills and medical knowledge they were expected to acquire over the rotation.

One significant limitation of this study is the low response rate, despite the incentive of a gift card for participating in the survey. In retrospect, the timing of the enrollment period over the holidays, when medical students may not be regularly on university email systems, may have contributed to poor enrollment. Unfortunately, it is therefore challenging to generalize the conclusions to the attitudes of all medical students in the class, institution, region, or more broadly.

Another limitation of this study is the fact that there is no control group against which to compare when considering pre-pandemic levels of stress and the quality of the OBGYN rotation prior to pandemic-related changes. It therefore may have been too speculative to ask students to compare their experience in their OBGYN clerkship to pre-pandemic experiences; however, the pandemic was an unexpected event, and as such, we did not have the opportunity to collect data prior to its onset.

Another limitation is the fact that this survey was distributed to all students at the same time. The time between finishing the OBGYN rotation and completing the survey was therefore not standardized across students, and some of the respondents may be prone to recall bias. Finally, this was a single-center study. A future direction to build upon could be that of investigating the OBGYN clerkship experience across various Canadian medical schools to get a broader perspective of the ways in which it has been impacted by the pandemic.

## Conclusions

The COVID-19 pandemic has had a significant effect on the OBGYN clerkship experience and student wellness at a single institution. This study has identified the need for increased accessibility of mental health resources to reduce stress and has highlighted vulnerable populations, such as those students interested in OBGYN while on this rotation. This study has highlighted OBGYN rotation quality issues, including reduced clinical volumes and decreased access to hands-on learning opportunities, with a particular gap identified in the access to gynecology learning opportunities. This study can be used as a source for clinical educators looking to adapt their OBGYN clerkship curriculum for future pandemics and provides valuable information on contingency plans for future circumstances where in-person OBGYN clerkship training may be limited.

## Appendices

Appendix A

Demographic:

1.     Age :

2.      Sex:

A)   Male

B)   Female

C)   Other:

D)   Prefer not to say

3.     Who are you currently residing with?

A) Alone

B) With parents

C) With partner

D) With partner and children

E) With children

F) With a room-mate

G) Other (specify): __________________

4.     Are you taking care of any dependents?

A) Yes (e.g. child, and/or an elderly person)

B) No

5. During your OB/GYN rotation did you participate in any of the following activities?

A)   Volunteering (e.g., call center, supporting staffs and residents, Public health office): Yes or No

a.     If yes, please describe:

B)   Remunerated work outside of clerkship: Yes or No

a.     If yes, please describe:

C)   Hobbies and self care activities: Yes or No

a.     If yes, please describe:

6. Prior to your rotation, were you considering a career in OB/GYN?

A) Yes

B) No

Stress

1.     Within the context of the COVID-19 pandemic, your stress level during clerkship was:

A)   Higher than usual

B)   Similar to usual

C)   Less than usual

2.     Within the context of the COVID-19 pandemic, your stress level during your OBGYN rotation compared to other rotations was:

A)    Higher than other rotations

B)    Similar to other rotations 

C)     Lower than other rotations

3.     How did Covid-19 impact your OBGYN Clerkship Experience POSITIVELY?

4.     How did Covid-19 impact your OBGYN Clerkship Experience NEGATIVELY?

5.     Has the current pandemic led you to reconsider your choice of residency program?

A) Yes

B) No

6.     If you answered "yes" to the previous question, which of the following statements best describes your situation?

A)    I have a greater interest in OB/GYN

B)    I have a lesser interest in OB/GYN

C)    None of the previous statements apply to me.

7.     Has the current pandemic led you to question your choice of medicine as a career?

A)   Yes

B)   No

8.     On a scale of 0 to 5, 0 being not stressful and 5 being an extreme level of stress, how did the following factors impact you during your OB/GYN rotation within the context of the COVID-19 pandemic?

**Table 12:** Potential sources of stress while completing your OB/GYN rotation during the COVID-19 pandemic

Potential sources of stress while completing your OB/GYN rotation during the COVID-19 pandemic	No stress				Extreme stress	Not Applicable
	1	2	3	4	5	
Fear of contracting COVID-19 or of contaminating someone else						
Suspension of OB/GYN clerkship rotations						
Modalities of rotations (e.g., lack of supervision, lack of learning opportunities)						
CaRMS applications						
Feeling the need to use this time to be productive in view of CaRMS						
Not having enough letters of recommendation or having letters of poorer quality						
CaRMS application deadline						
Cancellation of electives or not being able to book preferred electives						
Not being able to complete electives outside of your home university						

Quality of rotation

1.     Did you feel there was there a noticeable change in the quality of your OB/GYN rotation due to the COVID-19 Pandemic?

A)   Yes

B)   No

C)   If yes, please describe:

2.     Did you experience a lack of patients in the clinic due to the COVID-19 Pandemic?

A)   Yes

B)   No

3.     Do you feel your experience was impacted by providing virtual care vs. in person care?

A)   Yes

B)   No

C)   If yes, please describe:

4.     Do you feel you were limited in ability to learn hands-on skills (cervical exams, assisting in vaginal deliveries, etc.) due to the COVID-19 Pandemic?

A)   Yes

B)   No

C)   If yes, please describe:

5.     Did you experience reduced opportunities to participate in surgical procedures due to the COVID-19 Pandemic?

A)   Yes

B)   No

C)   If yes, please describe:

6.     Did you experience a lack of PPE at any point at your clinic?

A)   Yes

B)   No

7.     Do you feel your learning experience was affected by the provision of OB/GYN teaching seminars in a virtual fashion rather than in-person?

A)   Yes

B)   No

C)   If yes, please describe:

Well-being

**Table 13:** WHO Well-Being Index.

Over the last two weeks	All of the time (5)	Most of the time (4)	More than half of the time (3)	Less than half of the time (2)	Some of the time (1)	At no time (0)
I have felt cheerful and in good spirits.	5	4	3	2	1	0
I have felt calm and relaxed.	5	4	3	2	1	0
I have felt active and vigorous.	5	4	3	2	1	0
I woke up feeling fresh and rested.	5	4	3	2	1	0
My daily life has been filled with things that interest me.	5	4	3	2	1	0

Stress management and resources

1.     Have you used any university resources to overcome difficult moments during the COVID-19 pandemic; and if yes, did you find them helpful?

A) Yes, I have used university resources and I found them helpful.

B) Yes, I have used university resources, but I did not find them helpful.

C) No, I did not use any university resource.

2.     If you answered “Yes” to the previous question, what university resource(s) did you use?

A) Student support network

B) Student association

C) Members of your faculty 

D) Support line

E) Mobile application or other online resources

F) Other (specify) : _____________________

3.     Did you find university resources relating to stress and mental health easy to access during the COVID-19 Pandemic?

A)   Yes, university resources were easily accessed

B)   No, university resources were difficult to access

C)   I did not use university resources during the COVID-19 Pandemic
